# Immunofluorescent Evidence for Nuclear Localization of Aromatase in Astrocytes in the Rat Central Nervous System

**DOI:** 10.3390/ijms23168946

**Published:** 2022-08-11

**Authors:** Diána Kata, Ilona Gróf, Zsófia Hoyk, Eszter Ducza, Mária A. Deli, István Zupkó, Imre Földesi

**Affiliations:** 1 Department of Laboratory Medicine, Albert Szent-Györgyi Medical School, University of Szeged, 6725 Szeged, Hungary; 2 Department of Cell Biology and Molecular Medicine, Albert Szent-Györgyi Medical School, University of Szeged, 6720 Szeged, Hungary; 3 Institute of Biophysics, Biological Research Centre, 6726 Szeged, Hungary; 4 Department of Pharmacodynamics and Biopharmacy, Faculty of Pharmacy, University of Szeged, 6720 Szeged, Hungary

**Keywords:** aromatase, astrocyte, estrogen, estrogen receptor alpha, microglia, central nervous system

## Abstract

Estrogens regulate a variety of neuroendocrine, reproductive and also non-reproductive brain functions. Estradiol biosynthesis in the central nervous system (CNS) is catalyzed by the enzyme aromatase, which is expressed in several brain regions by neurons, astrocytes and microglia. In this study, we performed a complex fluorescent immunocytochemical analysis which revealed that aromatase is colocalized with the nuclear stain in glial fibrillary acidic protein (GFAP) positive astrocytes in cell cultures. Confocal immunofluorescent Z-stack scanning analysis confirmed the colocalization of aromatase with the nuclear DAPI signal. Nuclear aromatase was also detectable in the S100β positive astrocyte subpopulation. When the nuclear aromatase signal was present, estrogen receptor alpha was also abundant in the nucleus. Immunostaining of frozen brain tissue sections showed that the nuclear colocalization of the enzyme in GFAP-positive astrocytes is also detectable in the adult rat brain. CD11b/c labelled microglial cells express aromatase, but the immunopositive signal was distributed only in the cytoplasm both in the ramified and amoeboid microglial forms. Immunostaining of rat ovarian tissue sections and human granulosa cells revealed that aromatase was present only in the cytoplasm. This novel observation suggests a new unique mechanism in astrocytes that may regulate certain CNS functions via estradiol production.

## 1. Introduction

Sex steroid hormones such as estradiol and their receptors (ERs) have long been recognized for their important role in several brain functions such as synaptic transmission, glial differentiation, cognition, behaviour and the control of neurogenesis [1,2,3,4]. Estrogens also have a profound influence on the onset and progression of diseases that have sex differences in prevalence, such as Alzheimer’s disease, Parkinson’s disease or multiple sclerosis [5,6]. Both the peripheral and central nervous tissues are capable of synthesizing and metabolizing steroid hormones, including estrogens [1].

Aromatase (Aro), the rate-limiting enzyme in estrogen biosynthesis [7], is present in all vertebrates as the product of the single gene CYP19A1 in humans [8]. Aro activity and expression have been described in multiple brain regions such as the cerebral cortex, basal forebrain, hippocampus, thalamus or the cerebellum, and also in different cell types [9]. It is produced predominantly by neurons and also by glial cells such as astrocytes and microglia [5,9]. Crosstalk between astrocyte and microglia through secreted molecules such as cytokines, chemokines and estrogens modulate CNS immune responses in pathological conditions [10,11]. Increased Aro expression in reactive astroglia was found in response to brain injury [1]. Aro expression appears to be upregulated in astrocytes during the course of Alzheimer’s Disease [12]. Estrogens released by astrocytes play an important role in the compensatory restructuring of injured brain tissue [1]. Furthermore, even the neuroprotective effect of testosterone is partly thought to be related to its conversion to estradiol by Aro [2].

The presence and distribution of Aro in the CNS were demonstrated previously by using different techniques such as immunocytochemistry and immunohistochemistry with chromogen staining [13,14,15,16], fluorescent immunocytochemistry and immunohistochemistry [4,12,16,17,18] and imaging techniques with radiolabeled vorozole [19,20]. According to previous studies, Aro is demonstrated to be present in the entire cytoplasm, in the cytoplasm with a reticulated pattern (endoplasmic reticulum) and/or surrounding the nuclei or the axon terminals and dendritic spines of principal neurons; however, until now, nuclear localization of Aro was not reported. In this study, we present direct immunofluorescent evidence to show that Aro, a microsomal enzyme, uniquely colocalizes with the nucleus in primary astrocytes derived from newborn rats and also in adult rat brain tissue. We investigated the possibility of nuclear Aro expression in rat microglia cells in primary cell culture as well as in rat ovarian tissue and in human granulosa cells, which are the main sources of estrogen production during the reproductive age. Since the effects of estrogens are mediated by estrogen receptors (ERs), we also investigated the possible colocalization of nuclear Aro with estrogen receptor alpha (ERα) in the rat CNS.

## 2. Results

### 2.1. Nuclear Localization of Aromatase in Astrocytes

**Figure 1:**
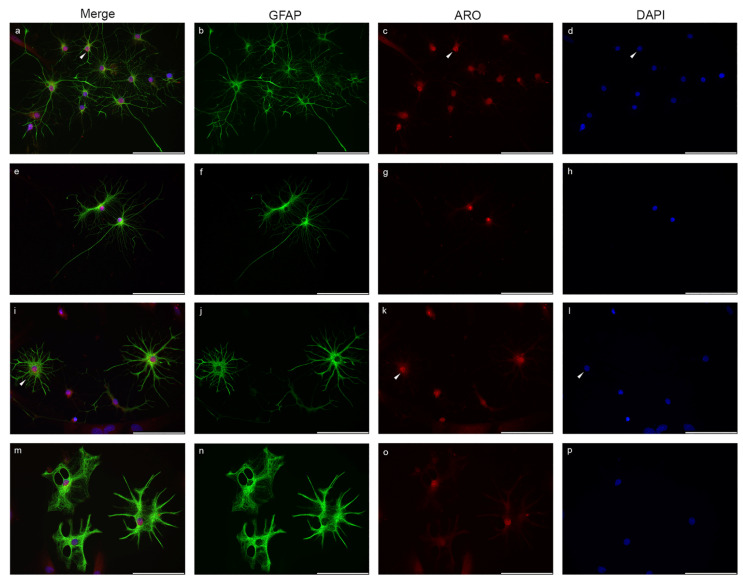
Aromatase (Aro) expression in astrocytes in glia-enriched subcultures. Cells were isolated from P0–P5 newborn rats. Aro (red) is strongly expressed in GFAP-positive astrocytes (green) and colocalizes with the nuclear staining (DAPI, blue). Fibrous astrocytes with small somata and long, thin unbranched processes (**a**–**h**) show strong nuclear Aro expression (**a**,**c**,**d**, white arrowheads). Cytoplasmic Aro expression is weaker, and the long thin processes do not show Aro immunopositivity. Protoplasmic astrocytes (**i**–**p**) with bigger somata and short, thick and frequently branched processes also display nuclear Aro expression (**i**,**k**,**l**, white arrowheads). Weak immunopositivity is detectable in the processes. The nuclear Aro signal distributes evenly or in a dotted pattern (**c**,**k**, white arrowheads) or strongly concentrates to certain parts of the nucleus (**g**) in both astrocyte subtypes. (White arrowheads indicate a selected representative cell and its nucleus with the Aro signal). Scale bar: 100 μm.

**Figure 2:**
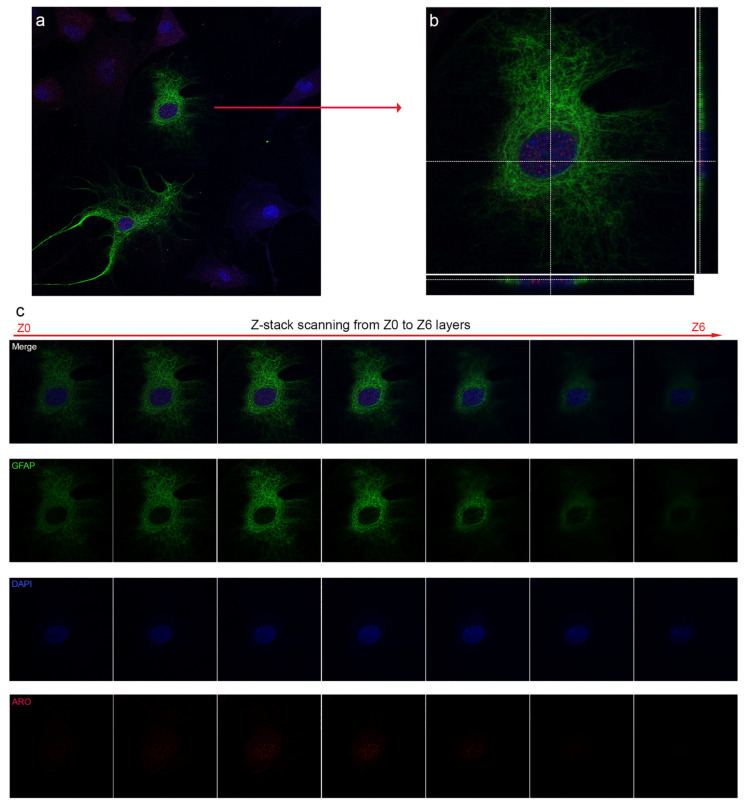
Confocal immunofluorescent analysis of GFAP positive astrocytes. Z-stack scanning analysis confirms Aro (red) immunopositivity in the nuclear layer. One representative cell with GFAP (green) and nuclear Aro immunopositivity (**a**) is selected for Z-stack scanning (**c**). The selected astrocyte shows dotted nuclear Aro positivity (**b**). Orthogonal scales from Z3 layer prove that the Aro signal originates from the nucleus (**b**) as the immunopositive dots colocalize with the nuclear signal (blue). Magnification: 60×.

Nuclear immunopositivity of Aro in different astrocyte subtypes from glia-enriched cell cultures is shown in Figure 1 and Figure 2. GFAP-positive astrocytes showed the strongest Aro immunopositivity in the nucleoplasm (Figure 1). Fibrous astrocytes with small somata and typical long, thin, smooth processes are shown in Figure 1a–h. In these cells, the Aro signal was extremely strong within the nucleus. The signal showed either an evenly strong distribution in the whole nucleus or certain parts of the nucleus or appeared in a dotted pattern (Figure 1c). The somata showed a weaker signal. Aro immunopositivity was not detectable in the long, thin processes. Protoplasmic astrocytes with thicker somata and short, thick, more branched processes are shown in Figure 1i–p. The Aro signal in these cells was also abundant in the nuclei and much weaker in the cytoplasm. The short, thick processes showed weak but detectable immunopositivity, unlike the fibrous processes.

To confirm the colocalization of Aro and the nuclear signal, we performed a confocal laser scanning analysis (Figure 2). GFAP-positive astrocytes with nuclear Aro signals are shown in Figure 2a, where both astrocytes showed dotted Aro positivity in the nucleoplasm. One representative cell was selected for further Z stack scanning analysis (Figure 2b,c). The merged picture from the Z3 layer with orthogonal scales (Figure 2b) proved that the Aro signal indeed originated from the nuclear layer and colocalized with the DAPI signal. The strongest Aro signals in the selected astrocyte were detected in the nuclear Z2–Z3 layers (Figure 2c).

**Figure 3:**
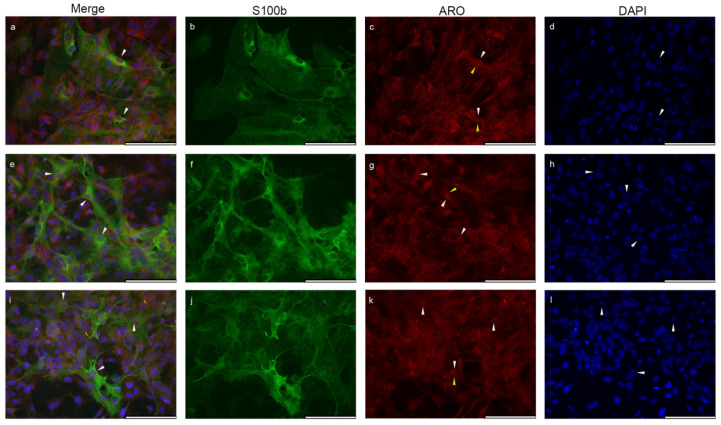
Nuclear colocalization of Aro in S100β positive cells from P0–P5 newborn rats. The three individual sets of pictures (**a**–**l**) show that Aro (red) is also expressed in S100β-positive astrocytes (green) in glial cell culture. Aro immunopositivity is detectable in the close perinuclear cytoplasmic area (**c**,**g**,**k**; yellow arrowheads) and also colocalizes (**a**,**c**,**e**,**g**,**i**,**k**; white arrowheads) with the nuclear signal (blue). Scale bar: 100 μm.

In the S100β positive subpopulation of astrocytes, Aro immunopositivity was also present in the nucleus (Figure 3) and in the perinuclear area, but compared to GFAP-positive cells, the signal appeared to be weaker.

**Figure 4:**
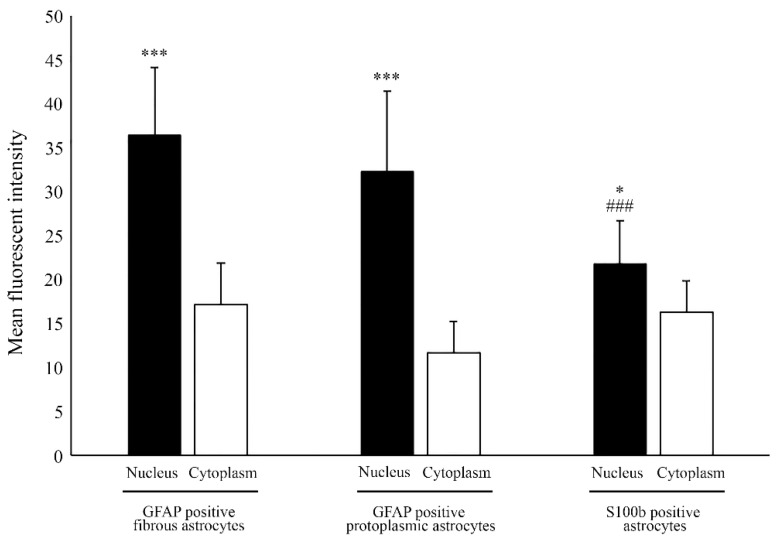
Quantitative analysis of aromatase localization in astrocytes. Analysis of mean fluorescent intensity revealed that Aro intensity is significantly stronger in the nucleus than in the cytoplasm in different astrocyte subtypes. GFAP-positive fibrous astrocytes showed stronger nuclear Aro intensity (36.36 ± 7.7; *n* = 25) than GFAP-positive protoplasmic astrocytes (32.25 ± 9.18; *n* = 29). Nuclear Aro intensity in the S100b-positive subtype (21.72 ± 4.98; *n* = 24) was significantly lower than in the GFAP-positive subtype. Data are presented in mean ± SD. * *p* < 0.05, *** *p* < 0.001, ^###^ *p* < 0.001. Asterisks indicate the significance between the nucleus and cytoplasm within each group. Pound (###) indicates the significantly lower intensity of nuclear Aro between GFAP and S100b positive astrocyte subtypes.

To quantify the differences in Aro localization, we measured the mean fluorescent intensity of the nucleus and the cytoplasm in GFAP-positive and also the S100b-positive astrocytes (Figure 4). The results showed that Aro immunopositivity was significantly stronger in the nucleus than in the cytoplasm. Fibrous astrocytes showed stronger nuclear Aro intensity (36.37 ± 7.7) than protoplasmic astrocytes (32.25 ± 9.18), but the difference was not significant. However, nuclear Aro intensity was significantly stronger in GFAP positive astrocyte subtype than in the S100b-positive subtype (21.72 ± 4.98).

**Figure 5:**
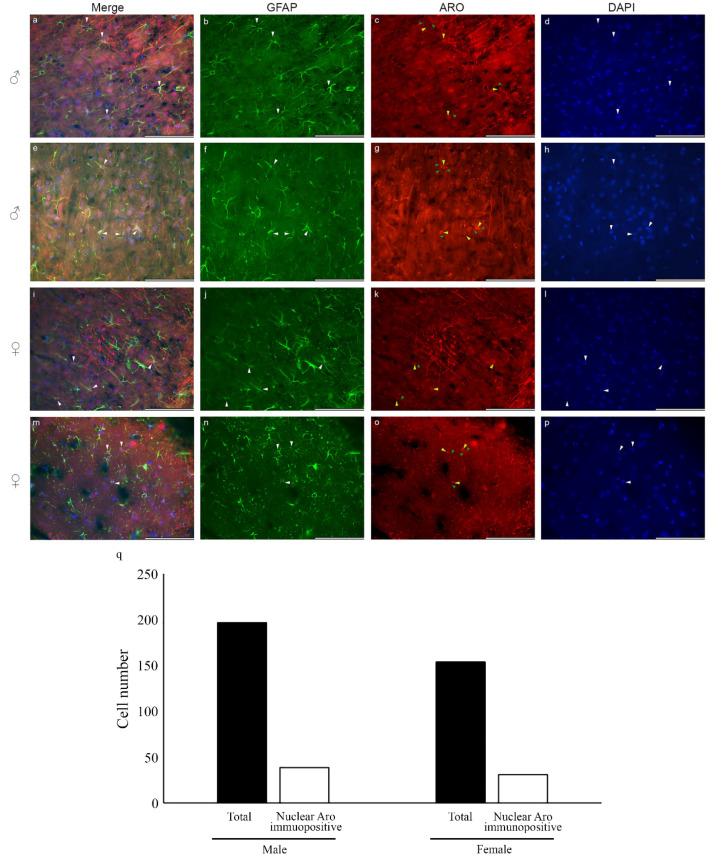
Localization of aromatase in astrocytes in adult rat brain sections. Nuclear (nucleus: blue) appearance of Aro enzyme (red; **c**,**g**,**k**,**o**; yellow arrowheads) in GFAP-labelled (green) astrocytes (**a**,**b**,**e**,**f**,**i**,**j**,**m**,**n**; white arrowheads) is also detectable in frozen cortical tissue sections from male (**a**–**h**) and female (**i**–**p**) rats. Interestingly, the Aro immunopositive signal in the adult cortical astrocytes is abundant in the processes (**c**,**g**,**k**,**o**; green arrowheads), while astrocytes derived from newborn rats do not show an Aro signal in the branches. Quantitative analysis of total GFAP-positive astrocytes and nuclear Aro immunopositive astrocytes (**q**) in 10 randomly selected microscopic fields of different frozen sections from both sexes showed that the abundance of nuclear Aro immunopositive GFAP-labelled astrocytes is about 20% in both males (19.79%; *n* = 197) and females (20.13%; *n* = 154). Scale bar: 100 μm.

Fluorescent immunohistochemistry on frozen tissue sections revealed that nuclear Aro is also detectable in the adult rat brain in both sexes. Colocalization of GFAP and Aro in male and female adult rat cortical astrocytes is shown in Figure 5a–p. Aro immunopositivity in frozen brain sections was presented in the nucleus and the cytoplasm and is also distributed in the processes. Quantitative analysis of nuclear Aro immunopositive astrocytes revealed that about 20% of the astrocytes show nuclear Aro immunopositivity in both sexes (Figure 5q).

### 2.2. Microglia Cells Only Show Cytoplasmic Aromatase Positivity

**Figure 6:**
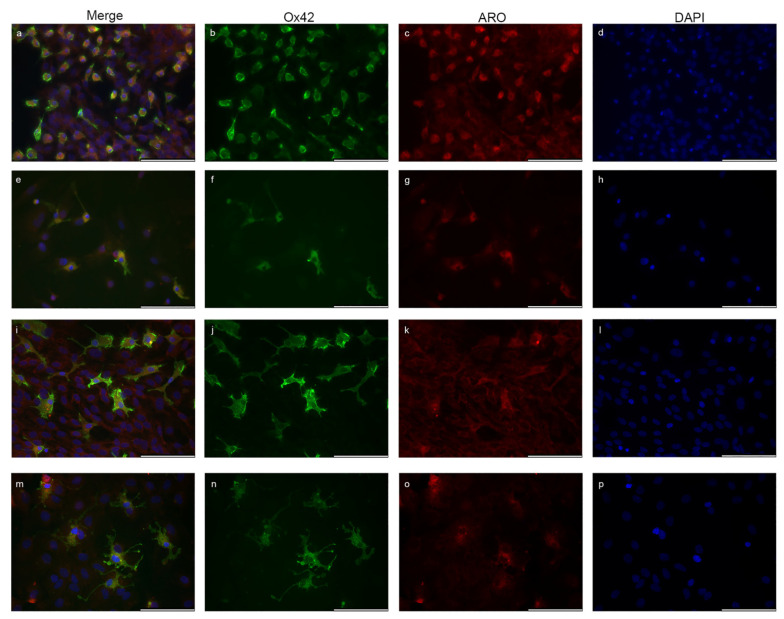

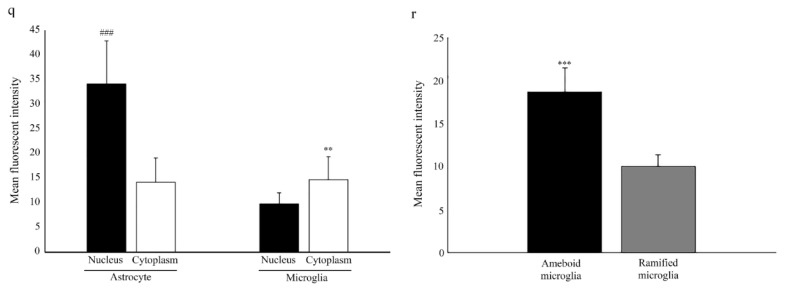
Localization of aromatase in microglia cells. CD11b/c (Ox42)-labelled microglia cells (green) isolated from newborn rats show Aro (red) expression, but the signal is detectable exclusively in the cytoplasm. Microglia cells with typical ameboid/activated morphology (**a**–**h**) show stronger Aro expression compared with the resting/ramified forms (**i**–**p**), where Aro immunopositivity is much weaker. Aro signal is weak or undetectable in the branches of ramified microglia cells. Quantitative analysis of the fluorescent intensity confirmed that the Aro signal is significantly higher in the cytoplasm of ameboid cells (18.64 ± 2.81; *n* = 15) than in ramified cells (9.98 ± 1.35; *n* = 15) (**r**). Although Aro is not presented in the nucleus of microglia cells, a low level of fluorescent intensity was detectable in the nuclear area due to the overprojection of staining of neighbouring cells from the upper and lower cellular layers and/or the overlapping of cytoplasmic signal, but its level was significantly lower (9.71 ± 2.31; *n* = 45) than the cytoplasmic Aro intensity (14.67 ± 4.68; *n* = 45). The analysis of mean fluorescent intensity also revealed that the level of Aro immunopositivity in the cytoplasm is similar in astrocytes (14.20 ± 4.9; *n* = 54) and microglia cells (**q**). Data are presented in mean ± SD. ** *p* < 0.01, *** *p* < 0.001, ^###^ *p* < 0.001. Asterisks indicate the significance between microglia cells. Pound (###) indicates the significance between astrocytes and microglia. Scale bar: 100 μm.

CD11b/c (OX42)-labelled microglia cells are shown in Figure 6. Aro expression was presented in the cytoplasm of the resting/ramified and also in the activated/ameboid microglia forms, but it was undetectable in the nucleus. Strong cytoplasmic Aro expression was connected with the ameboid morphology (Figure 6a–h). Resting/ramified microglia forms showed weaker Aro positivity in the cytoplasm, and the signal was undetectable or extremely weak in the processes and branches (Figure 6i–p). Quantitative analysis of mean fluorescent intensity showed that the level of Aro immunopositivity in the cytoplasm is similar in astrocytes (14.20 ± 4.9) and microglia (14.67 ± 4.68) cells (Figure 6q). Although Aro is not present in the nucleus of microglia cells, a low level of fluorescent intensity was detectable in the nuclear area due to the overprojection of staining of neighbouring cells from the upper and lower cellular layers and/or the overlapping of cytoplasmic signal, but it was significantly lower than the cytoplasmic Aro intensity. The analysis of fluorescent intensity between microglia cells revealed that activated/ameboid microglia cells showed significantly stronger cytoplasmic Aro intensity than the resting/ramified microglia cells (Figure 6r).

### 2.3. Estrogen Receptor Alpha Is Strongly Expressed in Astrocytes

**Figure 7:**
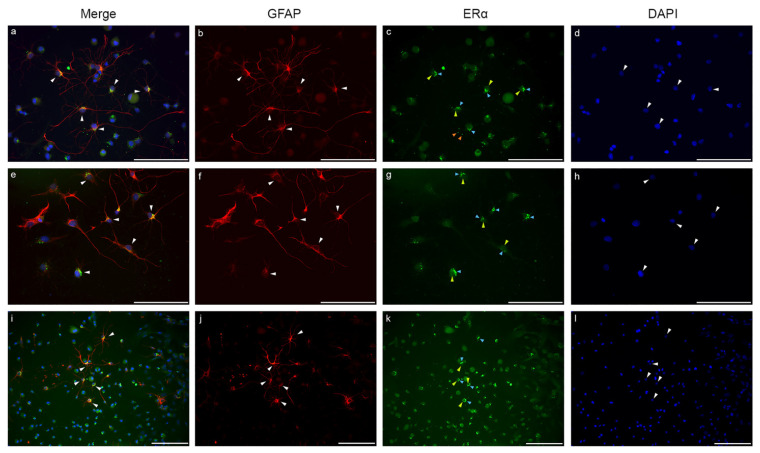
ERα is strongly represented in astrocytes. In GFAP (red)-labelled astrocytes (**a**,**b**,**e**,**f**,**i**,**j**; white arrowheads), ERα (green) is heavily expressed (**c**,**g**,**k**). The ERα signal in fibrous astrocytes shows strong and predominantly perinuclear localization in the cytoplasm (**c**,**g**,**k**; blue arrowheads), but immunopositive dots are also detectable in the processes (**c**; orange arrowheads). Nuclear ERα signals are represented as strong, unevenly distributed immunopositive dots or as a weak but even signal (**c**,**g**,**k**; yellow arrowheads). Nuclei are stained with blue (**d**,**h**,**l**). Scale bar: 100 μm.

The ERα distribution in GFAP-labelled astrocytes from glia-enriched subcultures is shown in Figure 7. ERα expression was detectable both in fibrous and protoplasmic astrocytes. Fibrous astrocytes showed stronger and more localized ERα immunopositivity (Figure 7a–l), while protoplasmic astrocytes showed weaker immunopositivity with evenly distributed patterns both in the cytoplasm and in the nucleus. In fibrous astrocytes, the ERα signal was predominant in the perinuclear cytoplasmic area (Figure 7c,g,k; blue arrowheads). Strong ERα immunopositive dots were also detectable in the fine processes (Figure 7c,g,k; orange arrowheads). The nuclear ERα signals were presented as strong, unevenly distributed immunopositive dots (Figure 7c,g,k; yellow arrowheads).

### 2.4. Colocalization of Nuclear Aromatase and Estrogen Receptor Alpha

**Figure 8:**
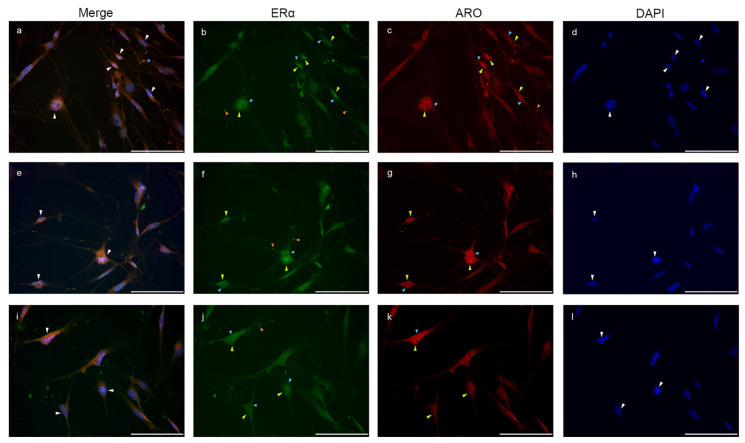
Nuclear aromatase expression overlaps with nuclear ERα signal. Double immunostaining with ERα (green) and Aro (red) in glia culture (**a**–**l**) shows that nuclear Aro signals colocalize with nuclear ERα (**a**,**e**,**i**; white arrowheads). Both Aro and ERα are strongly represented in the nucleus (**b**,**c**,**f**,**g**,**j,k**; yellow arrowheads) and also in the cytoplasm (**b**,**c**,**f**,**g**,**j**,**k**; blue arrowheads). ERα positive dots are also detectable in the processes (**b**,**f,j**; orange arrowheads). Scale bar: 100 μm.

Double immunostaining of Aro and ERα in glia-enriched subcultures is shown in Figure 8. The nuclear Aro immunosignal colocalized with the nuclear ERα signal. Both cytoplasmic and nuclear expression of Aro and ERα were detectable in the cells, and some immunopositive dots also appeared in the processes. When abundant nuclear Aro immunopositivity was detectable, ERα also showed much stronger immunopositivity in the nucleus than in the cytoplasm.

### 2.5. Nuclear Aromatase Expression Is Not Present in the Ovarian Cells and Tissue

**Figure 9:**
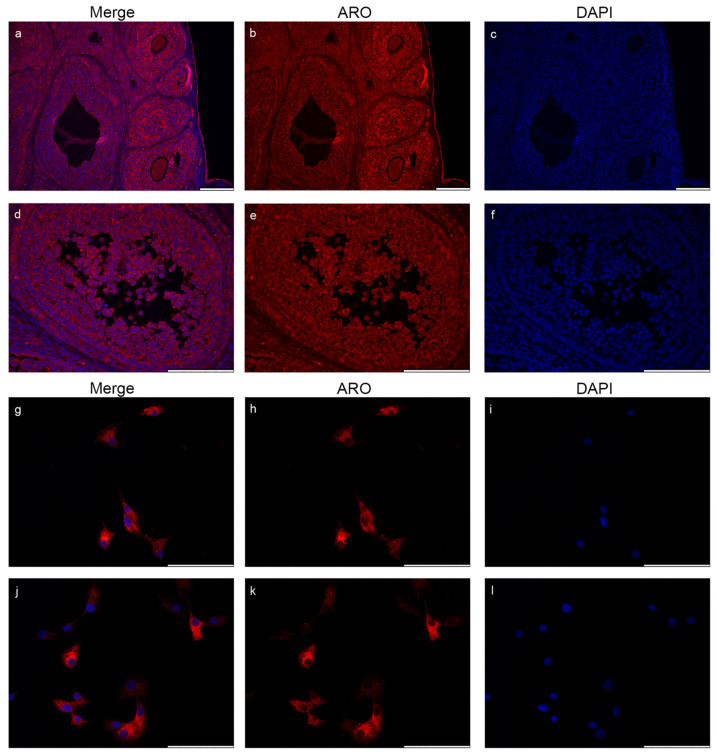
Localization of aromatase in the rat ovary and in human granulosa cells. Immunostaining of rat ovarian tissue sections is presented in images (**a**–**f**). Immunohistochemistry revealed that Aro (red) is strongly expressed in the whole ovarian tissue during the oestrus cycle but does not colocalize with the nuclear signal (blue). Results from Aro-stained human granulosa cells (red) corroborate the immunohistochemical findings as Aro immunopositivity is abundant in granulosa cells but only in the cytoplasm (**g**–**l**). Scale bar: 100 μm.

In order to validate the specificity of the Aro antibody used in our experiments and to examine the possibility of nuclear Aro expression in other tissues, we performed Aro immunostaining on paraffin-embedded rat ovary tissue sections (Figure 9a–f) and on human granulosa cell cultures (Figure 9g–l). Fluorescent immunohistochemistry revealed that Aro expression is strong in the whole rat ovary during the oestrous phase. Aro immunopositivity was distributed in the whole cytoplasm, but there was no sign of nuclear expression of the enzyme. Similarly, Aro immunopositivity was strongly present in the cytoplasm of human granulosa cells, but it was undetectable within the nucleus (Figure 9g–l).

## 3. Discussion

Aromatase, a product of the CYP19A1 gene, is the only enzyme responsible for the conversion of aromatizable androgens to estrogens in mammals [7,8]. The main sources of Aro activity are the gonads and adipose tissue, but there is an increasing body of evidence that Aro is present in the CNS too, where, apart from its classical effect on neuroendocrine functions and sexual behaviour, it plays an important role in the neuronal and glial differentiation, neuronal survivor and neuroprotection [1]. These beneficial effects are mediated by estradiol, the main product of Aro action.

In the CNS, Aro is predominantly expressed by neurons but also produced by glial cells [21]. Aro is constitutively expressed by certain astrocyte subpopulations [9]. Upregulated Aro expression and activity were observed in astroglia after brain injury [22,23,24] in all brain areas, and ER was also upregulated after neural damage [25].

The main finding of our study is the immunohistochemical evidence of the unique nuclear Aro localization in both fibrous and protoplasmic astrocytes in glia enriched subcultures. To our best knowledge, this is the first report of the nuclear localization of Aro. GFAP-labelled astrocytes derived from P0-P5 rats showed strong Aro co-expression with the nuclear signal. The cytoplasm of the somata showed weaker Aro immunopositivity, and only protoplasmic astrocytes presented weak Aro expression in the processes. Quantitative analysis of the mean fluorescent intensity confirmed that the Aro signal is significantly stronger in the nucleus than in the cytoplasm. Confocal laser scanning microscopy of GFAP-positive astrocytes confirmed the nuclear origin of Aro immunopositivity. Z-stack scanning with a thickness of 0.5 µm proved that the Aro and DAPI overlapping is intranuclear and not an overprojection of staining from the upper/lower cellular or cytoplasmic layers. As in the GFAP-labelled cells, nuclear Aro was similarly detectable in the S100β-positive subtype of cultured astrocytes, although the fluorescent intensity of Aro in the nucleus was significantly weaker in S100b-positive cells. The nuclear localization of Aro was also investigated by double immunostaining of frozen adult rat brain sections with GFAP and Aro. The presence of nuclear Aro was confirmed in both sexes in the adult rat brain. The abundance of nuclear Aro immunopositive GFAP-labelled astrocytes was about 20% in both males and females.

**Figure 10:**
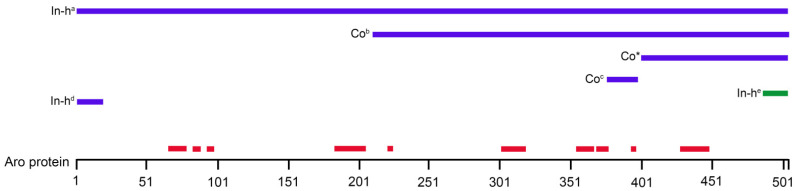
The simplified structure of human aromatase (Aro) protein with important conserved regions shown in red (based on Di Nardo et al. [8]). Blue lines indicate which parts of the human Aro protein were used to produce antibodies for Aro detection in CNS studies (Table 1). Green line indicates a mouse Aro peptide to generate an anti-mouse Aro antibody. Co: commercially available antibodies; In-h: antibodies made and validated in-house; * the Novus Aro antibody used in this study.

In the past few decades, multiple investigations tried to reveal the expressional pattern of Aro in the brain, but none of them found or mentioned the possibility of nuclear Aro expression. As we summarized in Table 1, there is a handful of studies that investigated the distribution of Aro in different brain areas and cell types. In these studies, multiple methods were used to detect Aro, although some of them were not suitable to determine the subcellular localization of the enzyme (e.g., positron emission tomography). The difference between our data and the previous findings could be related to differences in antibodies applied in other studies. The antibodies that were used to detect Aro in previous studies showed large diversity, but until now, none of the applied antibodies were able to detect Aro in the nucleus. All of the previously used Aro antibodies were raised against different residues of the aromatase peptide sequence, mostly of the human Aro protein (Figure 10), and multiple antibodies were made and validated in-house (Table 1). Previous studies also mentioned that detection of endogenous Aro protein could be difficult because of its low expression level and the limited sensitivity of the available antibodies [26]. In spite of the limited availability of commercially available aromatase antibodies for studying its expression in the rat brain by immunohistochemistry, as highlighted by Krentzel et al. [4], to date, the aromatase antibody from Novus Biologicals (B100-1596) was not applied for investigating Aro expression in the CNS. The Novus Aro antibody was first applied for studying the primate corpus luteum function [27,28] and peripheral blood leucocytes [29] by Western blotting. Recently, ovarian mitochondrial proteins [30] and the effects of glyphosate on ovarian folliculogenesis and steroidogenesis [31] were investigated by Western blotting and fluorescent immunohistochemistry. In our present report, we applied for the first time the Novus Aro antibody to investigate the localization of Aro in rat CNS.

It is important to mention that the human Aro gene is expressed in a cell- and tissue-specific manner [26]. This process is directed by tissue-specific promoters and an alternative first exon, called I.f., in the brain tissue. This type of regulation is crucial for the tissue-specific expression of Aro in normal and cancer-associated tissues [8,26]. Aro activity is also affected by phosphorylation, and some of the residues can undergo post-translational modification as well [8]. Phosphorylation is a rapid way to modulate enzyme activity in neurons in birds and also in mammals [8], which is also associated with important physiological and behavioural responses as a consequence of a rapid change in estrogen levels [33].

To demonstrate the specificity of the Novus Aro antibody applied in our study and to investigate the possible nuclear Aro distribution, we performed fluorescent immunostaining on rat ovarian tissue during the oestrus cycle and on human granulosa cells isolated from follicular fluid from patients undergoing IVF. As we expected, Aro was strongly expressed in the whole ovarian tissue and in the cytoplasm of human granulosa cells, but it was undetectable within the nucleus.

Local conversation of estrogens via the enzyme Aro has also been reported in microglia cells [5]. Estrogens play a major role in regulating microglia activity [5]. They have been reported to inhibit the inflammatory responses in microglia [34]. Estrogens have the ability to block the pro-inflammatory IL-1β and TNF-α synthesis and to increase anti-inflammatory IL-10 production [1,5]. Double immunostaining with microglia marker CD11b/c and Aro revealed that in our cultured microglia cells, both ramified and amoeboid microglia express Aro exclusively in the cytoplasm. Aro was not expressed in the cell nucleus. Activated/amoeboid microglia forms showed stronger Aro expression in the cytoplasm than resting microglia cells. Quantitative analysis confirmed that the fluorescent intensity of Aro is significantly higher in the cytoplasm of ameboid cells than in ramified cells. Aro immunopositivity was also not detectable in the branches of ramified cells.

The regulation of estrogen-mediated neuroprotection occurs via the activation of nuclear estrogen receptors (ERs) [4]. ERα and ERβ have been described to be expressed in neurons, astrocytes and microglia cells [1,35]. Furthermore, in spinal neurons, nociception modulated by estrogen occurs via classic nuclear ERs and also via plasma membrane-associated ERs [36]. Both ERα and ERβ can induce anti-inflammatory responses in microglia cells, although ERα is the more effective isoform [5]. ERs inhibit the production of pro-inflammatory cytokines via interfering with Toll-like receptor signalling [5]. ERα has also been reported to inhibit the pro-inflammatory NF-κB signalling in microglia and astrocytes [1,5] by impairing the translocation of NF-κB from the cytoplasm to the nucleus [34]. Since we demonstrated the nuclear localization of Aro in astrocytes, it raised the possibility of the co-existence of Aro and ERα within the nucleus. Therefore, we performed double immunostaining of GFAP and ERα in glia-enriched subcultures and also an Aro and ERα immunostaining in the same cultures to investigate the ERα distribution in astrocytes and also the feasibility of an overlapping nuclear Aro and ERα signal. Our results showed that ERα is widely expressed in GFAP-positive astrocytes. Protoplasmic astrocytes show weaker but evenly distributed ERα positivity in the cytoplasm and also in the nucleus. In fibrous astrocytes, strong ERα distribution was detectable in the perinuclear area. Immunopositive dots were detectable in the nucleus and also in the processes. We also demonstrated that when abundant nuclear Aro immunopositivity is visible in the cells, ERα also displayed stronger immunopositivity in the nucleus than in the cytoplasm.

Although the significance and the function of the nuclear presence of Aro in astrocytes are largely unknown, taking into consideration the important role of estrogens in neuroprotection and anti-inflammatory processes mediated by these cells, we could hypothesize that the nuclear expression of Aro allows the fastest local estrogen production and the immediate estrogen-derived response of astrocytes to inflammation or any other events having influence on these cell types. Astrocytes actively regulate CNS inflammation via reactive astrogliosis [37]. Estrogens inhibit the production of TNF-α and IL-6 in astrocytes [1]. In addition, the anti-inflammatory actions of estrogen in astrocytes result from nuclear liganded ERs and the suppression of NF-κB-dependent signalling [34,38]. In neurons, Aro is expressed in the perikarya, dendrites, synaptic terminals, presynaptic boutons and synaptic vesicles, which enables rapid estrogenic regulation of synaptic transmission, synaptogenesis and synaptic plasticity [4,9,39]. Immunoelectron microscopy by Hojo et al. [18] revealed that cytochromes are not only localized in the endoplasmic reticulum but also in both the axon terminals and dendritic spines of principal neurons. They are also located in presynaptic compartments as well as within the postsynaptic compartments. Later it was also found that membrane ERs and Aro not only co-express in the same spinal neuron, but also perform a multimeric signalling complex via oligomerization [39]. In the local presence of Aro, ERs on presynaptic terminals and dendritic membranes, Aro and ERs are able to form a unique signalling complex called “synaptocrine signalling” in rat spinal neurons [36]. This special estrogenic signalling type, where the synthesis and the action occur within the oligomer complex, allows unique subcellular modulation of synaptic estrogen concentration, which allows estrogen to act as an intracellular messenger [36,39]. The blood supply of the CNS could provide enough aromatizable androgen substrates for Aro to produce estrogens, but the local synthesis of such steroids is also possible since, e.g., hippocampal neurons are capable of synthesizing estradiol de novo from cholesterol [18]. Thus, in the course of the “synaptocrine signalling” process, the rapid and specific modulation of local estrogen concentration occurs independently of any other estrogen production. In addition, the possible role of Aro in converting molecules other than aromatization of androgens should not be excluded since it was demonstrated previously that Aro is able to catalyze other chemical reactions such as 1β- and 2β-hydroxylations of androgens, 2-hydroxylation of estrogens and, more interestingly, methylation of various substrates such as dopamine [40].

In summary, in this present report, we provided immunofluorescent evidence on the unique nuclear localization of Aro in astrocytes in cell cultures and also in adult brain sections in male and female rats. In contrast, Aro was detectable exclusively in the cytoplasm in microglial cells as well as in the ovarian tissue and cells. We also demonstrated the co-expression of Aro and ERα in astrocytes. Although the function and molecular mechanism of intranuclear Aro were not investigated in this study, we do believe that this piece of new information could help in better understanding the complexity of estradiol-related biochemical processes in the CNS. We hope that our findings may trigger further studies to reveal the possible clinical significance of this phenomenon.

## 4. Materials and Methods

### 4.1. Housing and Handling of the Animals

The animals were kept in accordance with the European Communities Council Directive (2010/63/EU) and the Hungarian Act for the Protection of Animals in Research (Article 32 of Act XXVIII). Sprague–Dawley rats (Animalab Ltd., Vác, Hungary) were kept at 22 ± 3 °C; the relative humidity was 30–70%, and the light/dark cycle was 12/12 h. The animals were maintained on a standard rodent pellet diet (INNOVO Ltd., Gödöllő, Hungary) with tap water available ad libitum. The rats were terminated by CO_2_ inhalation.

### 4.2. Preparation of Mixed Primary Cortical Cultures and Glia Enriched Subcultures

Mixed primary cortical cultures (*n* = 30) were established from neonatal (P0–P5) Sprague-Dawley rats by the method described earlier [41,42]. After decapitation, the frontal lobes of the cerebral cortex were collected, minced with scissors and dissociated with 0.25% trypsin (Gibco, Life Technologies, Carlsbad, CA, USA) in Dulbecco’s Modified Eagle Medium (DMEM, Gibco) containing 1g/L D-glucose, 2 mM L-glutamine and pyruvate supplemented with 1x antibiotic-antimycotic solution (Gibco) for 10 min at 37 °C in a humidified air atmosphere supplemented with 5% CO_2_. The tissue suspensions were transferred into conical tubes and centrifuged at 1000× *g* for 10 min at room temperature. The pellets were resuspended in DMEM containing 10% FBS (Biowest, Riverside, MO, USA) then the cells were separated with repeated pipetting. The dissociated pellets were centrifuged at 1000× *g* for 10 min at room temperature. The final pellets were resuspended in 5 mL DMEM/10% FBS and the cells were plated on Advanced TC cell culture flasks (72 cm^2^, 12 × 10^6^ cells/flask; Greiner Bio-One International GmbH, Kremsmünster, Austria) and cultured at 37 °C in a humidified air atmosphere supplemented with 5% CO_2_ for seven days in vitro (DIV).

Glia-enriched subcultures were prepared from the mixed primary cultures (DIV7) by shaking the flasks at 150 rpm in a platform shaker for 30 min at 37 °C. Glial cells from the supernatant were collected and centrifuged at 3000× *g* for 8 min at room temperature. The pellets were resuspended in 2 mL of DMEM/10% FBS. Cells were seeded at a density of 2 × 10^5^ cells in poly-L-lysine (Sigma-Aldrich, St. Louis, USA) coated coverslips (18 × 18 mm) and cultured for another 6 days in DMEM/10% FBS at 37 °C in a 95% air/ 5% CO_2_ atmosphere.

### 4.3. Preparation of Human Granulosa Cell Culture

Luteinized granulosa cells were collected from follicular fluid obtained from patients undergoing oocyte retrieval for in vitro fertilization (IVF). Granulosa cell preparation was performed with modifications of the protocol that was previously described [43]. The collected follicular fluids were centrifuged at 100× *g* for 3 min. The pellets were resuspended and washed three times in phosphate-buffered saline (PBS) containing 1% FBS, then incubated with 20 IU/mL hyaluronidase (Sigma-Aldrich) for 12 min at 37 °C in a humidified air atmosphere supplemented with 5% CO_2_. After incubation, the cell suspensions were washed, and granulosa cells were separated by gradient centrifugation. Cells were layered on 40% Percoll/PBS and centrifuged at 2000× *g* for 10 min to separate the granulose cell from other cellular components (e.g., blood cells). Granulosa cells were aspirated from the interface and washed with a cell culture medium (Medium 199 Earle’s, Biochrom GmbH, Berlin, Germany) containing 100 μg/mL gentamycin, 2 mM L-glutamine and 10% FBS. The final pellets were resuspended in a 1 mL cell culture medium. Granulosa cells were seeded at a density of 1 × 10^5^ cells/coverslip (18 × 18 mm) and cultured for 3 days (DIV3) in Medium 199/10% FBS at 37 °C in a humidified air atmosphere supplemented with 5% CO_2_.

### 4.4. Immunocytochemistry

Glia-enriched subcultures and granulosa cell cultures were fixed on coverslips with 4% formaldehyde for 10 min and rinsed twice with 0.05 M phosphate-buffered saline (PBS, pH: 7.4) for 5 min. After permeabilization and blocking for 30 min at 37 °C in 0.05 M PBS containing 5% normal goat serum (NGS), 1% bovine serum albumin (BSA) and 0.05% TritonX-100, cells were incubated overnight at 4 °C in a humidified chamber with the appropriate primary antibodies listed in Table 2. Cells were washed four times with 0.05 M PBS for 10 min, then incubated with the following secondary antibodies for 3 h: Alexa Fluor 568 conjugated anti-rabbit IgG (1:1000; Invitrogen, Waltham, Massachusetts, USA, #A11011), Alexa Flour 488 conjugated anti-mouse IgG (1:1000; Invitrogen, #A11001). Cells were washed four times with 0.05 M PBS for 10 min. Nuclei were stained, and the cells were mounted on microscope slides with Prolong Diamond Antifade Mountant medium with DAPI (Invitrogen, #P36962).

### 4.5. Immunostaining of Frozen Brain Tissue Sections

Brain tissues were collected from mature (200 g) male and female Sprague-Dawley rats after intracardial perfusion with 0.9% sodium chloride dissolved in 0.01 M phosphate buffer (PB, pH 7.4) followed by 4% paraformaldehyde in 0.1 M PB (pH 7.4). After perfusion, the brains were postfixed for 2 h in the same fixative, then washed in 0.1 M PB (pH 7.4) and cryoprotected in 30% sucrose solution at 4 °C until saturation. Sagittal sections of the brain with a thickness of 15 μm were cut on a cryostat and then transferred onto glass slides. For fluorescent immunostaining, the frozen tissue sections were rehydrated with washing buffer (PBS pH 7.4). To block, non-specific staining sections were incubated in blocking buffer (5% NGS in PBS) for 1 h at room temperature. Primary antibodies (Table 2) were applied in the appropriate dilution in incubating buffer containing 1% BSA, 1% NGS and 0.4% Triton-X100 in PBS and incubated overnight at 4 °C. Sections were washed three times for 10 min with washing buffer and then incubated with fluorescent secondary antibodies diluted in incubating buffer for 2 h at room temperature. Sections were washed twice for 10 min with washing buffer and once for 10 min with distilled water. Nuclei were stained, and the slides were mounted with coverslips and Prolong Diamond Antifade Mountant medium with DAPI (Invitrogen). Negative technical control staining on three different samples was also made by the same method but omitting the primary rabbit-anti Aro antibody (see in Supplementary Materials; Figure S1).

### 4.6. Immunostaining of Formalin-Fixed, Paraffin-Embedded Ovary Tissue Sections

Ovary tissues were collected from mature (10 weeks old, 180–200 g) female Sprague-Dawley rats. The oestrous cycle of the rats was confirmed by Electronic Monitor for Vaginal Estrous-cycle in Rat /Muromachi MK-12 (Animalab Ltd.), and vaginal secretion was observed using microscopy. Freshly dissected ovary tissues were fixed with 10% formalin dissolved in Sorensen’s phosphate buffer. After the standard paraffin-embedding procedure, 3 μm thick ovary sections were made from the embedded tissue blocks and then transferred onto glass slides. Immunohistochemistry was performed similarly as previously described [49]. For fluorescent immunostaining, paraffin-embedded tissue sections were deparaffinized in xylene, then transferred into 100% alcohol 3 times, then once to 95%, 70% and 50% ethanol and finally to distilled water. To perform antigen retrieval, slides were put into a staining container filled with 0.01 mM citrate buffer (pH: 6) and incubated at 95 °C for 20 min. After incubation, slides were washed 3 times with phosphate-buffered saline (PBS, pH 7.4) and then blocked for 1 h at room temperature with blocking buffer (PBS containing 5% NGS). Sections were then incubated overnight at 4 °C in a humidified chamber with primary antibodies (Table 2) diluted in the blocking buffer. After washing with PBS (3 × 10 min), samples were incubated with the appropriate fluorescent secondary antibodies for 2 h at room temperature. Sections were washed 2 × 10 min with PBS and once for 10 min with distilled water. Nuclei were stained, and the slides were mounted with coverslips and Prolong Diamond Antifade Mountant medium with DAPI (Invitrogen).

### 4.7. Image Analysis and Statistics

Fluorescent digital microscope images were acquired with Leica DMLB microscope and Leica DFC700T camera (Leica Microsystems GmbH, Wetzlar, Germany) or with Nikon Eclips Ti-U (Nikon Europe BV, Amsterdam, the Netherlands) and Andor Zyla VCS-09300 camera. Specific objective lens (Leica PL APO 20×/NA = 0.8 and PL APO 40×/NA = 0.75 or Nikon S Plan Fluor ELWD 40×/NA = 0.6) and LasX version 3.7.2 software (Leica Microsystems) or NIS-Elements BR 5.21.02 (Nikon) were used to capture images. Z stack images were taken with Leica TCS SP5 confocal laser scanning microscope (Leica Microsystems). Specific objective lens (HC PL APO 63×/NA = 1.4) and Las AF Lite version 3.1.0 were used during the scanning of the Z-axis sections with a thickness of 0.5 μm. A total of 400 fluorescent pictures were captured: from astrocytes *n* = 160 (including GFAP [*n* = 80], S100b [*n* = 60], Z-stack [=20]; from microglia cells *n* = 110; from ERα *n* = 60; from granulosa cells *n* = 30; from tissue sections *n* = 40. Pictures were further processed (for the assembly of figure panels) using Adobe Photoshop version 23.4.1. Mean fluorescent intensity for the quantitative analysis was performed by ImageJ 1.53k (NIH, Bethesda, MD, USA). All statistical comparisons were made by R Studio 1.3.1073. Results were analyzed with one-way ANOVA, and the Bonferroni correction was used to establish significance between groups. Values were presented as mean ± SD.

## Figures and Tables

**Table 1:** Summary of studies investigating aromatase expression in the CNS.

Species	Cell Type/Area	Localization of Aro	Method	Reference
Rat (Sprague-Dawley, adult, male, female)	Neurons/hypothalamus Neurons/subcortical telencephalic areas	Granular immunoreactivity in the somata and axon-like processes, but no staining in dendrites in hypothalamic cells Homogeneous but patchy labelling in the somata and dendritic processes in the subcortical areas *Glia cells were immunonegative!* There was no sexual dimorphism in the distribution and intensity of the staining **No indication of nuclear localization**	Immunostaining (**^a^** purified polyclonal rabbit-anti human placental Aro; **In-house antibody**)	[13]
Rat (Wistar, adult, male)	Pyramidal neurons/CA1-CA3 Granule cells/dentate gyrus Astrocytes/stratum radiatum and oriens Oligodendrocytes/hippocampus	Cell body, dendrites in CA3 pyramidal neurons, axon terminals and dendritic spines of principal cells, pre- and postsynaptic compartments Most of the glia cells were lacking P450arom immunoreactivity **No indication of nuclear localization**	Immunohistochemistry (**^a^** anti-P450arom IgG; **In-house antibody**) Postembedding immunogold labelling	[18]
Rat (Wistar, adult, male)	Neurons/olfactory bulb	Immunoreactivity mainly in the somata and also in cellular processes in juxtaglomerular neurons Weaker immunoreactivity in the cytoplasm (surrounding the nuclei) of mitral/tufted cells **No indication of nuclear localization**	Immunohistochemistry *(***^b^** rabbit anti-ARO polyclonal antibody-epitope corresponding to amino acids 209–503 mapping at the C-terminus of human CYP19. **Commercial antibody**, Santa Cruz Biotechnology; Antibody registry ID: AB_2088681)	[16]
Rat (Sprague-Dawley newborn, adult, male, female)	Nucleus caudatus/putamen Nucleus accumbens core and shell	Immunopositivity in the cytoplasm but empty nucleus No sexual dimorphism in the Aro immunopositivity **No indication of nuclear localization**	Immunohistochemistry (**^c^** monoclonal anti-aromatase, Residue 376–390 human p450, clone H4, **Commercial antibody**, BioRad; Antibody registry ID: AB_566942)	[4]
Human (male, female)	Neurons and glia cells/cholinergic basal forebrain nuclei, hypothalamic nuclei	Immunoreactivity in the processes and the soma in granular cytoplasmic pattern accumulating around the nucleus and near plasmalemma (staining the endoplasmic reticulum) in neurons Immunopositivity in glia cells (astrocytes, oligodendrocytes, ependymal and choroid plexus cells) but no data about the subcellular localization **No indication of nuclear localization**	Immunohistochemistry (**^d^** polyclonal rabbit antibody raised against the 20 amino acid peptide coupled to thyroglobulin by glutaraldehyde corresponding to the N-terminus of human Aro; **In-house antibody**)	[14]
Human (male, female)	Neurons/lateral temporal neocortex Astrocytes/ lateral temporal neocortex	Immunoreactivity in the perikaryon and proximal processes Immunoreactivity located in the soma and processes **No indication of nuclear localization**	Immunohistochemistry (antibody A: rabbit polyclonal AB recognizes human, rat, bovine, mouse, chicken Aro. **Commercial antibody**, Acris Antibody) (**^e^** antibody B: rabbit polyclonal AB generated from 15 amino acid peptic corresponding to residues 488–502 of mouse Aro. **In-house antibody**)	[15]
Human (male, female)	Whole brain	Distribution volume values followed the rank order: thalamus > amygdala > preoptic area > medulla > cortex > putamen > cerebellum > white matter No information about the subcellular distribution of aromatase **No indication of nuclear localization**	Positron emission tomography (PET) (with radiolabelled aromatase inhibitor[N-methyl-^11^C]vorozole)	[20]
Japanese quail (adult, male)	Neurons/hindbrain	Immunoreactive perikarya with immunonegative nuclei in neurons Immunoreactive fibres with punctate structures were also observed in neurons **No indication of nuclear localization**	Immunohistochemistry (polyclonal affinity-purified rabbit-anti quail recombinant antibody; **In-house antibody**)	[32]

**Table 2:** List of primary antibodies used in this study. According to the technical information provided by the manufacturers, the commercial primary antibodies used in the study were verified by Knockdown or Relative expression to ensure that the antibody binds to the antigen stated.

Name/Order Number (Manufacturer) Antibody Registry ID	Host/Clonality	Immunogen	Reactivity	Dilution	Citation
**Aromatase** **NB100-1596** (Novus Biologicals) AB_10000919	Rabbit/Polyclonal	C-terminal portion of the human aromatase protein (between residues 400–502)	Human, Mouse, Rat, Primate, Bovine, Rabbit	1:100	[31]
**CD11b/c (OX42)** **MA1-90756** (Thermo Scientific/Invitrogen) AB_2280688	Mouse/Monoclonal	Rat peritoneal macrophages	Rat	1:100	[44]
**Estrogen receptor alpha** **MA1-310** (Thermo Scientific/Invitrogen) AB_325422	Mouse/Monoclonal	Synthetic peptide corresponding to the residues E(247) V G M M K G G I R K D R R G(261) of the ER alpha DNA binding domain	Bovine, Human, Human, Mouse, Primate, Rat	1:100	[45]
**Glial fibrillary acidic protein (GFAP)** **MA5-12023** (Thermo Scientific/Invitrogen) AB_10984338	Mouse/Monoclonal	Glial fibrillary acidic protein	Chicken, Human, Pig, Rat	1:100	[46]
**Glial fibrillary acidic protein (GFAP)** **PA1-10019** (Thermo Scientific/Invitrogen) AB_1074611	Rabbit/Polyclonal	Full-length recombinant human GFAP protein	Bovine, Horse, Human, Mouse, Pig, Rat	1:1000	[47]
**S100β** **287 011** (**Synaptic Systems**) AB_2814881	Mouse/Monoclonal	Recombinant protein corresponding to AA 1 to 92 from rat S100B	Mouse, Rat	1:600	[48]

## Data Availability

Not applicable.

## Supplementary Materials

The following supporting information can be downloaded at: https://www.mdpi.com/article/10.3390/ijms23168946/s1.
