# Supplementary material for: Immunofluorescent Evidence for Nuclear Localization of Aromatase in Astrocytes in the Rat Central Nervous System
Source: Int J Mol Sci. 2022 Aug 11;23(16):8946. doi: 10.3390/ijms23168946 (PMC9408820; doi:10.3390/ijms23168946)
Supplement: Supplementary file 1 [file ijms-23-08946-s001.zip › ijms-1828741-supplementary.pdf]

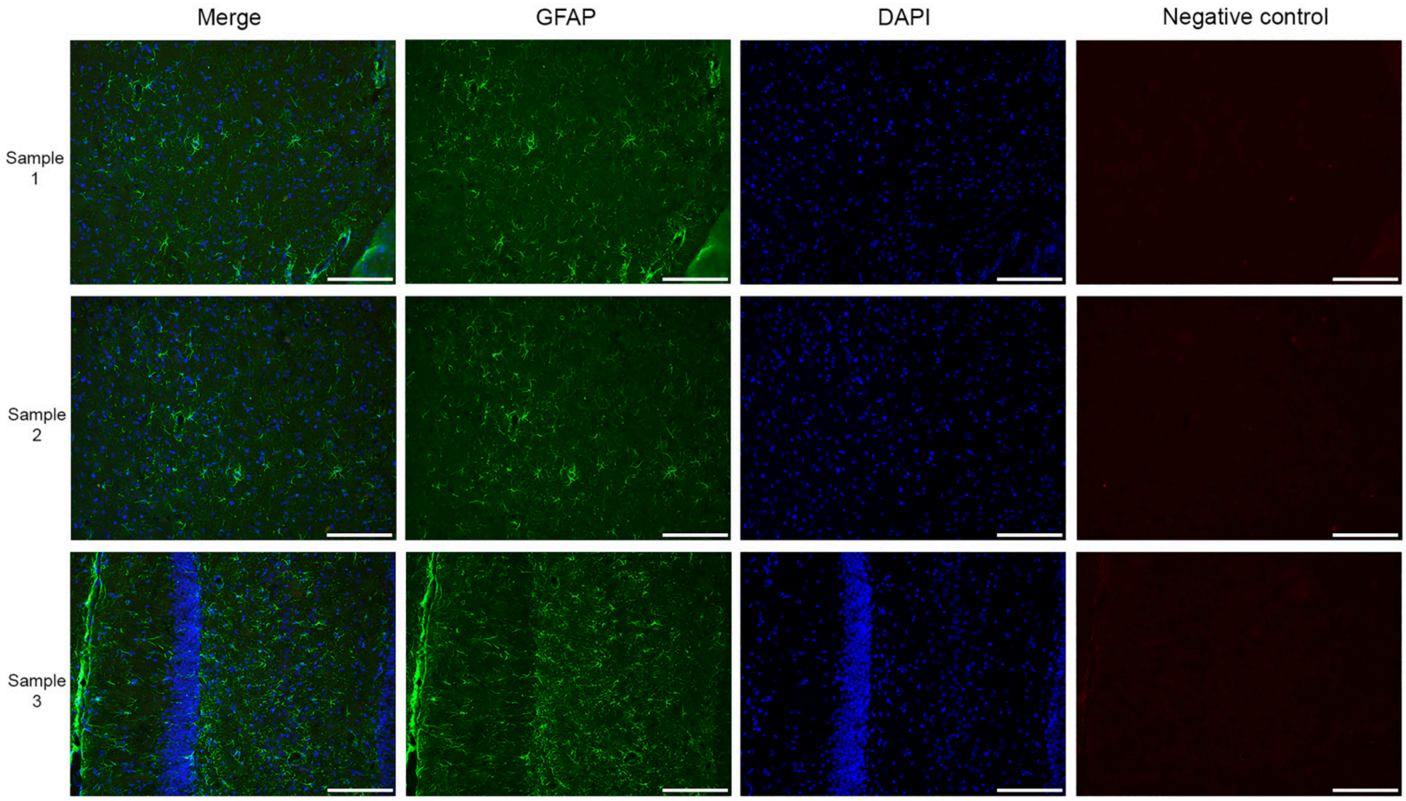

Figure S1. Negative control staining of frozen brain tissue section. Immunofluorescent staining on three different frozen samples performed with the method described in the materials section but omitting the primary rabbit-anti Aro antibody. The immunostaining revealed that without the appropriate primary Aro antibody there is no significant background signal which is caused by the secondary Alexa Fluor 568 conjugated anti-rabbit IgG itself. Green: GFAP labeled astrocytes; Blue: Nuclei. Scale bar: 100 µm
